# Root traits for infertile soils

**DOI:** 10.3389/fpls.2013.00193

**Published:** 2013-06-11

**Authors:** Philip J. White, Timothy S. George, Lionel X. Dupuy, Alison J. Karley, Tracy A. Valentine, Lea Wiesel, Jane Wishart

**Affiliations:** ^1^The James Hutton InstituteInvergowrie, UK; ^2^School of Biology, University of St. AndrewsSt. Andrews, UK

**Keywords:** root architecture, mineral nutrition, rhizosphere, soil solution, uptake

## Abstract

Crop production is often restricted by the availability of essential mineral elements. For example, the availability of N, P, K, and S limits low-input agriculture, the phytoavailability of Fe, Zn, and Cu limits crop production on alkaline and calcareous soils, and P, Mo, Mg, Ca, and K deficiencies, together with proton, Al and Mn toxicities, limit crop production on acid soils. Since essential mineral elements are acquired by the root system, the development of crop genotypes with root traits increasing their acquisition should increase yields on infertile soils. This paper examines root traits likely to improve the acquisition of these elements and observes that, although the efficient acquisition of a particular element requires a specific set of root traits, suites of traits can be identified that benefit the acquisition of a group of mineral elements. Elements can be divided into three Groups based on common trait requirements. Group 1 comprises N, S, K, B, and P. Group 2 comprises Fe, Zn, Cu, Mn, and Ni. Group 3 contains mineral elements that rarely affect crop production. It is argued that breeding for a limited number of distinct root ideotypes, addressing particular combinations of mineral imbalances, should be pursued.

## Introduction

Crop production, worldwide, is restricted by the concentrations and chemical forms of mineral elements present in the soil solution (Table 1). Adequate supplies of the essential mineral elements nitrogen (N), potassium (K), phosphorus (P), calcium (Ca), magnesium (Mg), sulphur (S), boron (B), iron (Fe), manganese (Mn), zinc (Zn), copper (Cu), nickel (Ni), molybdenum (Mo), and chlorine (Cl) are required for maximal crop production (White and Brown, 2010). Phytoavailability of N, K, P, or S often limits low-input agriculture (Fageria et al., 2011; Mueller et al., 2012), the phytoavailability of Fe, Zn, and Cu limits crop production on alkaline and calcareous soils, which comprise 25–30% of agricultural land (White and Broadley, 2009), and the phytoavailability of K, P, Mg, Ca, or Mo can limit crop production on acid soils, which comprise >40% of agricultural land (von Uexküll and Mutert, 1995; Sumner and Noble, 2003) and additionally suffer from excessive concentrations of protons, aluminium and Mn (Fageria et al., 2011; White and Greenwood, 2013). Since mineral elements are acquired by the root system, it has been suggested that the development of genotypes with appropriate root traits might increase crop yields on infertile soils (White et al., 2005, in press; Lynch, 2007, 2011, in press; Hawkesford, 2011). This paper examines the root traits likely to improve the acquisition of essential mineral elements. It deals with these traits in a general way that is not intended to provide parameter values for sophisticated mathematical models of resource capture (cf. Barber, 1995; Jungk and Claassen, 1997; Dunbabin et al., 2003; Roose and Fowler, 2004; Postma and Lynch, 2012). It is observed that, although efficient acquisition of a particular element requires a specific set of root traits, suites of traits can be identified that benefit the acquisition of several mineral elements. Breeding for a limited number of distinct root ideotypes, benefitting the acquisition of several elements coincidentally, would optimize the use of research resources.

**Table 1:** **Physical processes likely to supply essential mineral elements to the root surface of plants growing in the field, and the occurrence of deficiency disorders and mineral toxicity symptoms in agricultural systems**.

	**Physical process**	**Deficiency disorders**		**Toxicity symptoms**	
N	Mass flow ≥ Diffusion >> Interception	Frequent	Insufficient N supply	Occasional	Overapplication of N fertilizer
K	Diffusion > Mass flow >> Interception	Frequent	Low phytoavailability, especially in acid soils	Rare	
P	Diffusion >> Mass flow >> Interception	Frequent	Low phytoavailability, especially in acid soils	Rare	
Ca	Mass flow >> Diffusion ≈ Interception	Rare	Insufficient Ca in highly weathered tropical soils; low phytoavailability in strongly acidic, sodic or saline soils; low phytoavailability to horticultural crops	Occasional	Calcareous soils
Mg	Mass flow ≥ Diffusion >> Interception	Occasional	Insufficient Mg in shallow, coarse soils; low phytoavailability in calcareous, strongly acidic, saline or sodic soils	Rare	
S	Mass flow ≥ Diffusion >> Interception	Frequent	Insufficient S supply and low phytoavailability of S in organic fractions	Rare	
B	Mass flow >> Diffusion ≈ Interception	Frequent	Insufficient B in sandy, alkaline and heavily limed soils in high rainfall environments	Frequent	Sodic soils
Fe	Mass flow > Diffusion ≈ Interception	Frequent	Low phytoavailability in well aerated alkaline and calcareous soils	Occasional	Waterlogged soils
Mn	Mass flow > Diffusion ≈ Interception	Occasional	Insufficient Mn in coarse-textured, sandy soils; low phytoavailability in organic, alkaline and calcareous soils	Frequent	Acid mineral soils and waterlogged soils
Zn	Mass flow > Diffusion ≈ Interception	Frequent	Low phytoavailability in alkaline and calcareous soils	Occasional	Anthropogenically contaminated soils
Cu	Mass flow > Diffusion ≈ Interception	Frequent	Low phytoavailability in organic, alkaline and calcareous soils	Occasional	Anthropogenically contaminated soils
Ni	Mass flow >> Diffusion ≈ Interception	Rare	Low phytoavailability in alkaline and mineral soils	Occasional	Soils overlying serpentine or ultrabasic rocks; Anthropogenically contaminated soils
Mo	Mass flow >> Diffusion ≈ Interception	Rare	Low phytoavailability in acid soils	Rare	
Cl	Mass flow > Diffusion >>Interception	Rare	Leached soils with low Cl deposition rates	Frequent	Saline soils
Al			Not essential	Frequent	Acid soils
Na			Not essential	Frequent	Saline and sodic soils

It should be noted that the application of mineral fertilizers and soil amendments, soil type, chemistry and microbiology, and the prevailing environmental conditions will all influence the dominant physical process supplying essential mineral elements to the root surface. References: Barber (1995), White and Broadley (2001, 2003, 2009), Chapin et al. (2002), Oliveira et al. (2010), White and Brown (2010), Moradi et al. (2010), Brown and Bassil (2011), Fageria et al. (2011), Marschner and Rengel (2012), White and Greenwood (2013).

## The phytoavailability of essential mineral elements

If essential mineral elements are not present in the soil, they must be provided to enable crop production. Various agronomic strategies can be employed to increase the efficiency with which inorganic and organic fertilizers are used. In principle, these optimize the chemistry, quantity, placement, and timing of fertilizer applications (Fageria et al., 2011; Simpson et al., 2011; Mueller et al., 2012; White et al., 2012, in press; White and Greenwood, 2013). These agronomic strategies can be complemented by cultivating genotypes with appropriate root traits. When mineral elements are present in the soil, strategies can be developed to increase their acquisition by roots, thereby improving the mineral nutrition of crops and, ultimately, crop yields (Lynch, 2007, 2011, in press; White and Broadley, 2009; Richardson et al., 2011; White et al., 2012, in press; White and Greenwood, 2013).

Root traits improving the acquisition of essential mineral elements, and tolerance of potentially toxic mineral elements in the soil, have been the focus of many theoretical studies and field, glasshouse and laboratory investigations (White et al., 2005, in press; Lynch, 2007, 2011, in press). The availability of mineral elements for acquisition by roots is determined by (1) interception through root growth, (2) local diffusion in the rhizosphere, and (3) mass flow in the soil solution to the root surface (Table 1; Barber, 1995; Chapin et al., 2002; Fageria et al., 2011). Direct root interception is not considered to be important for the acquisition of mineral elements because the amounts required for plant nutrition are generally far greater than those intercepted. However, root proliferation and elongation are important in exploiting the soil volume, reducing the path length for diffusion and mass flow, and providing an extensive surface area for the uptake of mineral elements. Diffusion of mineral elements is determined by the concentration gradient between the soil solution and the root surface (White and Greenwood, 2013). It operates over short distances, such as the width of the rhizosphere, and is especially important for the acquisition of P and K (Table 1). It is facilitated by agricultural practices and crop genotypes that increase the concentrations of mineral elements in the soil solution and high-capacity systems for their uptake by root cells (Lynch, 2011; White, 2013). The mass flow of a mineral element to the root surface is determined by its concentration in the soil solution and the transpiration-driven movement of water to the root (White and Greenwood, 2013). It is important for the acquisition of mineral elements with high concentrations in the soil solution, such as N, K, S, Ca, Mg and Cl in agricultural soils, and for mineral elements that are required in relatively small quantities by plants, such as Fe, Mn, Zn, Cu, Ni, B, and Mo (Table 1).

**Figure 1:**
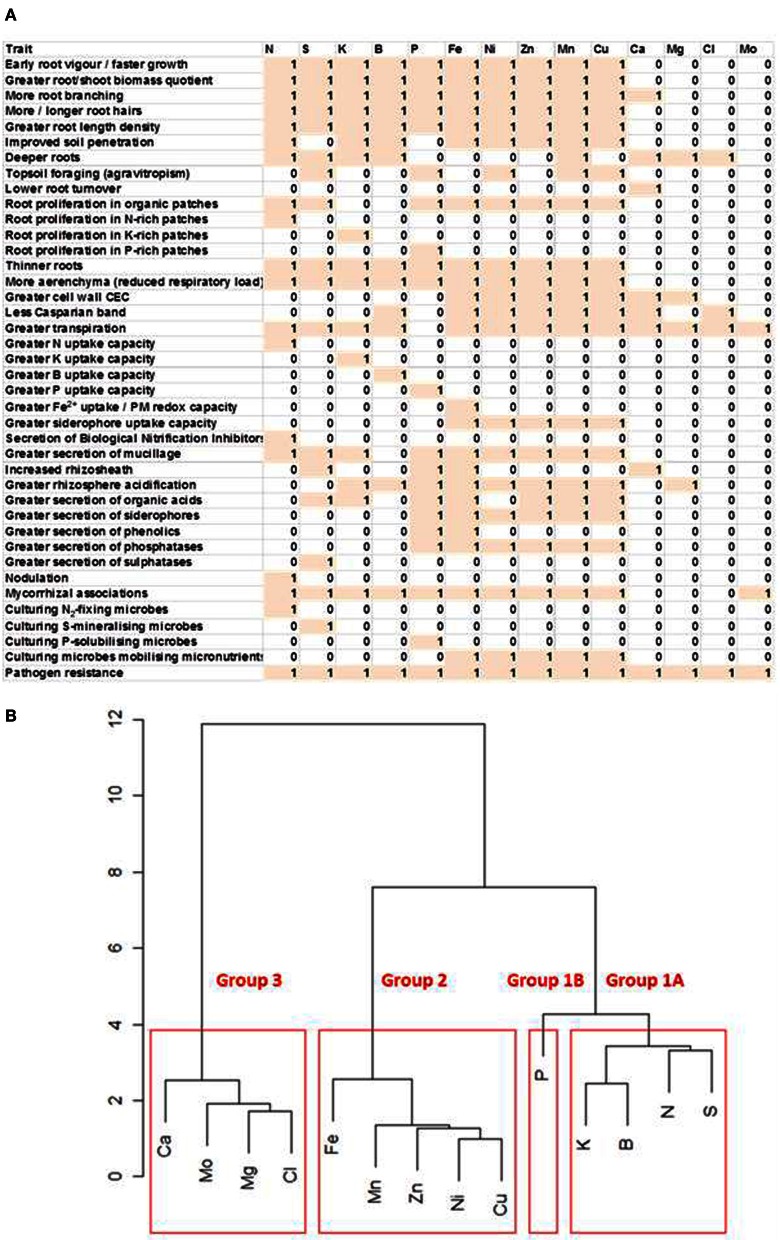
**(A)** Matrix of root traits likely to improve the acquisition of essential mineral elements. Traits are scored as being likely (1) or unlikely (0) to improve the acquisition of an essential mineral element in reduced-input agricultural systems. **(B)** Clustering of essential mineral elements requiring similar root traits to improve their acquisition. Relationships were calculated from the data presented in the matrix.

Root traits influencing the acquisition of mineral elements include: (1) root elongation rate, lateral root production, root hair characteristics, root length density (root length/soil volume) and soil penetration, all of which increase the volume of soil explored by the root system and the surface area for the uptake of mineral elements, (2) the gravitropism of root growth, which influences the ability to exploit different soil horizons, (3) the proliferation of roots in patches of soil containing high concentrations of mineral elements that are immobile in the soil, which reduces the carbon and energy requirement for their acquisition, (4) the turnover of fine roots, which redistributes carbon following the capture of localized resources, (5) specific root length (length/mass quotient) and formation of aerenchyma, which affect the carbon and energy requirement for resource capture by influencing root respiration, (6) high-capacity systems for the uptake of elements whose delivery to the root surface is determined by diffusion in the rhizosphere, (7) modification of rhizosphere pH and the exudation of organic solutes and enzymes, which affect the concentrations of mineral elements in the soil solution either directly through soil chemistry or indirectly though the culture of appropriate microbial communities, and (8) interactions with microbes either intimately, through mycorrhizal associations or nodulation, or remotely, through the culture of beneficial microbes or exclusion of pathogenic ones in the rhizosphere (Figure 1A).

## Root ideotypes for improving the acquisition of essential mineral elements

The acquisition of different mineral elements, and often different chemical forms of mineral elements, requires different root traits (Figure 1A). Nevertheless, suites of traits can be identified that benefit the acquisition of groups of mineral elements (Figure 1B). Since different chemical forms of mineral elements are interconverted in the soil, and crop mineral nutrition requires only the acquisition of an essential mineral element, root traits that benefit the acquisition of an element, rather than a particular chemical form of that element, have been identified. For example, the choice of traits for N acquisition is predicated on the supposition that nitrate is often the dominant chemical form of N in agricultural systems, but can be immobilized in the rhizosphere as ammonium, or converted to organic compounds through biological activities. Group 1 comprises elements that are required by crops in large amounts and are often in short supply in low-input agricultural systems. Group 1A comprises elements that are readily soluble and, therefore, can be present at high concentrations in the soil solution and follow the movement of water in the soil profile. This group includes N, when present as nitrate, and S, when present as sulphate, which are both delivered to the root surface primarily by mass flow, and K, which reaches the root surface both by mass flow and diffusion in the rhizosphere. Boron is also included in Group 1A. The only element in Group 1B is P, which is required in large quantities by plants, can be present in high amounts in the soil, but reaches the surface of the root primarily by diffusion. Group 2 comprises elements required in smaller amounts by crops that are often present in adequate amounts in soils, but whose phytoavailability is constrained by soil chemical properties, such as high soil pH. This group includes Fe, Zn, Ni, Mn, and Cu. Group 3 comprises elements whose supply rarely limits crop production, namely Ca, Mg, Cl, and Mo (White and Broadley, 2001, 2003; Brown and Bassil, 2011; White and Greenwood, 2013). Designing root ideotypes for Group 3 elements is not an immediate priority, and is not discussed further.

### Root ideotypes for the acquisition of nitrogen, sulphur, potassium, and boron

The Group 1A root architectural ideotype can be illustrated by the “steep, cheap, and deep” ideotype that facilitates nitrate acquisition, and N-fertilizer use efficiency, in crops (Lynch, in press; White et al., in press). Nitrate is present in high concentrations in the soil solution and follows the movements of water in the soil profile. Deeper roots are considered beneficial for nitrate acquisition and restricting the movement of nitrate to watercourses through leaching (Dunbabin et al., 2003; Lynch, in press; White et al., in press). Nitrate is delivered to plant roots predominantly by mass flow of the soil solution and nitrate acquisition can be accelerated by increasing root length and surface area, and by increasing transpiration (Garnett et al., 2009; Lynch, in press; White et al., in press). Increasing root nitrate uptake capacity can also improve N-acquisition (Lynch, in press). Lynch (in press) has argued that the proliferation of roots in patches of local abundance might be maladaptive for the acquisition of mineral elements that are readily soluble in the soil solution, such as nitrate. However, the proliferation of roots in N-rich patches can improve the acquisition of ammonium (NH^+^_4_) and organic N-compounds (see below). Increasing the abundance of cortical aerenchyma can reduce the carbon and energy costs associated with a large root system (Lynch, in press). Relationships with N_2_-fixing bacteria, whether symbiotic or associative, can improve the N-nutrition of crops and genotypes of both legumes and non-legumes fostering greater biological N_2_-fixation often have higher yields in N-limited environments (Kraiser et al., 2011; James and Baldani, 2012; Kumar et al., 2012). The N nutrition of plants growing on unfertilized soils can also be improved through mycorrhizal associations (Fitter and Moyersoen, 1996; Bennett et al., 2013).

Although nitrate is the form of N taken up by roots of most crops, they can also take up NH^+^_4_, urea, amino acids, and peptides (Miller and Cramer, 2005; Gojon et al., 2009; Kraiser et al., 2011). Ammonium is the main form of N taken up by plants adapted to acidic and anaerobic soils (Miller and Cramer, 2005). The root architectural ideotype for the acquisition of NH^+^_4_, which is relatively immobile in the soil, resembles that for the acquisition of K^+^. This ideotype incorporates high root length densities, which reduce the distance NH^+^_4_ must diffuse in the rhizosphere to reach the root surface, and the proliferation of roots in NH^+^_4_-rich patches. Plants releasing inhibitors of biological nitrification into the rhizosphere limit the conversion of NH^+^_4_ to nitrate and, thereby, retain N in the vicinity of the root (Subbarao et al., in press). This can improve the efficiency of N-fertilizer use by minimizing nitrate leaching.

Crops take up most of their S as sulphate (De Kok et al., 2011). Effective acquisition of sulphate, which is readily soluble and follows the movement of water in the soil profile, requires similar root architecture to the acquisition of nitrate (White et al., in press). In response to S-deficiency, plants generally increase their capacity for sulphate uptake and alter their root architecture to increase the volume of soil explored at depth, for example by the development of lateral roots close to root apices (López-Bucio et al., 2003; Gojon et al., 2009; De Kok et al., 2011). Increasing transpiration accelerates the delivery of S to the root surface. About 75–90% of the S in soils is present in the topsoil as organic compounds, and S phytoavailability can be enhanced by secreting enzymes degrading soil organic-S, either from plant roots themselves or from their associated microbial communities (Canfield and Farquhar, 2012; Lamers et al., 2012).

Potassium acquisition can be increased by accelerating K^+^ delivery to the root surface by increasing mass flow of the soil solution or K^+^ diffusion in the rhizosphere. Although K^+^ concentrations in soil solutions generally lie between 0.1 and 1 mM, this is not sufficient to supply a rapidly growing crop with enough K by mass flow alone. Root traits that improve K acquisition include: (1) early root vigor and the preferential partitioning of biomass to roots, (2) greater lateral rooting and production of root hairs, increased root length/mass quotient and increased root length density, all of which increase the surface area for K^+^ uptake by roots and reduce the distance required for K^+^ diffusion and water flow, (3) greater root penetration of strong soils, to improve access to soil resources, (4) the release of organic acids that solubilize “non-exchangeable” K in soils and increase the K^+^ concentration in the soil solution, (5) greater K^+^ uptake by root cells, which reduces the K^+^ concentration at the root surface and accelerates the delivery of K^+^ by diffusion, and (6) increasing transpiration, which increases the delivery of K^+^ to the root surface through mass flow of the soil solution (Jungk and Claassen, 1997; Rengel and Damon, 2008; White, 2013).

Boron is also included in Group 1A. Boron deficiency in crops is prevalent across a wide range of climates, cropping systems, and soils (Brown and Bassil, 2011). Like other Group 1A elements, B is readily soluble in water and is delivered to the root surface by mass flow. It is easily leached from soils and its distribution in the soil profile is determined by water movements. Thus, root architectural traits required for efficient B acquisition resemble those required for the efficient acquisition of N, S, and K. The phytoavailability of B is restricted in acid and mildly alkaline soils.

### Root ideotype for phosphorus acquisition

A “topsoil foraging” root architectural ideotype has been proposed for the acquisition of P, which is relatively immobile in the soil and concentrated in the topsoil. This ideotype incorporates: (1) early root vigor and the preferential production of roots in the topsoil, (2) greater root branching and the production of long root hairs, (3) high root length density in the topsoil and the proliferation of lateral roots in P-rich patches, (4) greater root length/mass quotient, either through the development of thinner roots or the formation of root aerenchyma, and (5) the partitioning of a greater proportion of plant biomass to the root system (White et al., 2005, in press; Lynch, 2007, 2011; White and Hammond, 2008; Richardson et al., 2011; Brown et al., in press). This architectural ideotype can be complemented by accelerating the diffusion of P to the root surface by increasing (1) the phosphate uptake capacity of root cells, and (2) the phosphate concentration in the rhizosphere solution through the secretion of protons and organic acids to solubilize P-salts and phytases and phosphatases to degrade organic P-compounds, which can be effected either by roots themselves or through the activities of beneficial microbes (Barea et al., 2005; White and Hammond, 2008; Richardson et al., 2011). Associations with mycorrhizal fungi are also known to improve plant P nutrition and increase crop yields (Smith and Read, 2008).

### Root ideotypes for elements with restricted phytoavailability in alkaline soils

Topsoil foraging is an appropriate root ideotype for the acquisition Mn, Cu, and Ni, which are relatively immobile in the soil and concentrated in the topsoil, but not necessarily for Fe and Zn, which are distributed more evenly and likely to be phytoavailable throughout the soil profile (White and Greenwood, 2013). An even spread of roots throughout the soil is more suitable for the acquisition of Fe and Zn. Since Fe, Zn, Mn, Cu, and Ni all have restricted mobility in the soil, their acquisition can be improved by investing more biomass in the root system, by developing a more extensive root system, and by proliferating lateral roots in mineral-rich patches (White and Broadley, 2009; White and Greenwood, 2013). Since the delivery of these elements to the root surface is largely determined by mass flow of the soil solution, increasing transpiration will accelerate their acquisition (White and Greenwood, 2013). Root traits increasing the phytoavailability of these elements in the rhizosphere, such as the secretion of protons, phytosiderophores, and organic acids, increases their acquisition by crops (White and Broadley, 2009, 2011; White and Greenwood, 2013). Similarly, the secretion of enzymes, such as phosphatases, able to degrade organic compounds that chelate cations can increase their phytoavailability and acquisition by crops (White and Broadley, 2009; White and Greenwood, 2013). Associations with mycorrhizal fungi, and the cultivation of microbes that increase the phytoavailability of these elements in the rhizosphere are also beneficial (Barea et al., 2005; White and Broadley, 2009; White and Greenwood, 2013). The diffusion of these elements in the rhizosphere to the root surface can be accelerated by increasing the capacity for their uptake by root cells, either as cations or as phytosiderophore complexes (White and Broadley, 2009; White and Greenwood, 2013). Increasing Fe(III) reductase activity in the rhizosphere increases Fe acquisition (White and Broadley, 2009) and the presence of microbes oxidizing Mn to Mn^2+^ increases Mn acquisition (Nogueira et al., 2007).

## Conclusions

Although the efficient acquisition of a particular mineral element requires a specific set of root traits, suites of traits can be identified that benefit the acquisition of groups of mineral elements (Figure 1B). Elements can be divided into three groups. The elements in Group 1 are nutrients that are often deficient in low-input agriculture. Group 1A comprises N, S, K, and B, whose inorganic forms are readily soluble in the soil solution and large amounts of which reach the root surface by mass flow. Group 1B includes only P, which reaches the root surface primarily by diffusion. The elements in Group 2 are the micronutrients Fe, Zn, Cu, Mn, and Ni that are often present in adequate amounts in soils, but whose phytoavailability is restricted in alkaline soils.

Several root traits will improve the acquisition of mineral elements generally (Figure 1). Breeding for crops with improved resource acquisition might target these traits for maximum effect. These traits include early root vigor and greater biomass allocation to roots, architectural traits that increase root exploration of the soil, and anatomical traits that reduce the respiratory burden. Mycorrhizal associations and resistance to root pathogens also appear to benefit the acquisition of most essential mineral elements. Increasing transpiration will improve the acquisition of all elements delivered to the root surface primarily by mass flow of the soil solution. The acquisition of a smaller set of elements is targeted by breeding for root proliferation in the topsoil (P, Mn, Cu, and Ni) or at depth (N, S, K, and B). Breeding for root proliferation in patches of organic matter will also improve the acquisition of several elements and benefits from the secretion of enzymes that degrade organic compounds into these areas. The secretion of mucilage, organic acids, and phytosiderophores will also improve the acquisition of several mineral elements. By contrast, breeding for root traits that increase the acquisition of either a single or a limited number of element(s) will have utility only in environments in which agricultural production is restricted by lack of a single or few key elements. Such traits include root proliferation in patches of soil with high phytoavailability of a specific element, increasing the phytoavailability of a single element either through the release of specific exudates or by culturing a microbial population with an exclusive function, and increasing the root uptake capacity for a single element.

We believe that a rational way to apportion resources for harnessing root traits for sustainable agriculture is to prioritize traits benefitting the acquisition of several essential mineral elements and pay less attention to those benefitting the acquisition of only one. In this manner root ideotypes for multiple environments can be produced.

### Conflict of interest statement

The authors declare that the research was conducted in the absence of any commercial or financial relationships that could be construed as a potential conflict of interest.
